# The association of monthly, diurnal and circadian variations with suicide attempts by young people

**DOI:** 10.1186/s13034-017-0171-6

**Published:** 2017-08-01

**Authors:** Türkan Akkaya-Kalayci, Nestor D. Kapusta, Thomas Waldhör, Victor Blüml, Luise Poustka, Zeliha Özlü-Erkilic

**Affiliations:** 1 Outpatient Clinic of Transcultural Psychiatry and Migration Induced Disorders in Childhood and Adolescence, Department of Child and Adolescent Psychiatry, Medical University of Vienna, Währinger Gürtel 18-20, 1090 Vienna, Austria; 2 Department for Psychoanalysis and Psychotherapy, Medical University of Vienna, Währinger Gürtel 18-20, 1090 Vienna, Austria; 3 Department for Epidemiology, Medical University of Vienna, Kinderspitalgasse 15/I, 1090 Vienna, Austria; 4 Department of Child and Adolescent Psychiatry, Medical University of Vienna, Währinger Gürtel 18-20, 1090 Vienna, Austria

**Keywords:** Suicide attempts, Youth, Adolescents, Diurnal and circadian changes, Turkey

## Abstract

**Background:**

Different psychosocial factors might have an impact on suicidal behaviour and evidence shows that there may be an association between monthly, diurnal and circadian changes and suicidal behaviours.

**Methods:**

In the present study we analysed retrospectively records of 2232 youth, who were treated in emergency units of state hospitals in Istanbul/Turkey after attempting suicide.

**Results:**

The majority of the suicide attempters were females (81.6%). In both sexes, suicide attempts most frequently occurred at the beginning of the calendar week and between evening and midnight.

**Conclusions:**

This study shows that suicide attempts in youth follow diurnal and circadian changes. As suicide attempts of youth most frequently occurred at the beginning of the calendar week and between evening and midnight, health services such as specialized counselling for youth should especially be available during this time.

## Background

Suicidal behaviour is an increasing public health problem worldwide [1–3]. In the last decades the suicide rates of adolescents aged between 15 and 19 increased faster than for other age groups [4–7] so that suicide is the second leading cause of death among adolescents in Europe [4, 8, 9]. However, suicide attempts as acts of intending to end one’s life [10] are observed much more frequently than completed suicides [3]. Among youth, the ratio of suicide attempts to completed suicides is approximately 25:1, whereas among adults it is about 4:1 [11]. Suicidal behaviour can be related to individual, biological and a number of psychosocial factors [12–14]. The evidence is growing that diurnal and circadian changes might have an influence on completed suicides [15] as well as on suicide attempts [16, 17]. Previous studies show that completed suicides among adults occur more frequently on Sundays [18] and Mondays [19, 20]. Similarly, among adolescents up to age 19, both attempted and completed suicides are observed more frequently at the beginning of the week on Mondays and Tuesdays [15]. Studies on adults show that suicide attempts occur most frequently in the evening and late evening [8, 21, 22] while completed suicides most frequently occur in the late morning [22–25]. Although suicidal behaviour is increasing among youth [4, 5, 7, 9] studies focusing on the diurnal and circadian rhythm of suicide attempts among adolescents and young adults are rare.

The aim of this study was to assess the monthly, diurnal and circadian rhythm of suicide attempts among youth. In accordance with the literature for adults we hypothesized that suicide attempts among youth are more frequent in the evenings and more common at the beginning of the week.

## Methods

The present study retrospectively analyses records of 2232 young people, who were treated after a suicide attempt in emergency units of state hospitals in Istanbul/Turkey between 1 January 2010 and 31 December 2010. Data were extracted from a standardized form, which is filled out by the medical staff in all cases of medical treatment after a suicide attempt, this is standard procedure in all emergency units of state hospitals in Istanbul. For the present study we obtained the data from the Statistics of Health Directorate of Istanbul at the Ministry of Health of Turkey, where the filled forms from state hospitals in Istanbul are collected. In the present study we analysed all suicide attempts by young people up to age 25 in 1 year.

The standardized questionnaire, which is used for the present study, was prepared by an expert team of the Ministry of Health of Turkey, for their own statistical survey. These standardized questionnaires were filled out by the medical staff, who had treated the patient after a suicide attempt. This standardized questionnaire comprises items about date, time, methods and reasons for suicide attempts, sociodemographic profiles (e.g. age, sex, marital, educational and occupational status), physical and mental health problems, drugs anamnesis and psychiatric treatment. The medical staff filled out the required items in the questionnaire, after conducting a detailed medical history with the patient. The present study is based on the records of these standardised questionnaires and analysed retrospectively all the records of all emergency units of all 39 state hospitals in Istanbul, therefore our study sample refers to the whole area of Istanbul.

For the present study we analysed the association between suicidal behaviour and diurnal as well as circadian and monthly changes.

Daily counts of suicides were analysed by fitting a Poisson regression model in SAS 9.4 (SAS Institute Inc. (2012). SAS Institute Inc., 2002–2012. Cary, NC, USA) using “*proc genmod*”. Because of over-dispersion the option *pscale* was used.

Independent variables were treated as class variables and were as following: hour (reference = 00:00 hour), weekday (reference = Monday) and sex (reference = female). Population-at-risk estimates were not available and therefore assumed to be constant during the whole time interval. The number of daily counts was used as the dependent variable in the regression model. For each combination of the variables: hour, weekday, month and sex, i.e. 24*7*12*2 groups, daily counts of cases were calculated.

Inclusion of all four independent variables simultaneously was not possible because of over-parameterization due to the large number of subgroups based on the four variables. Therefore three bivariable regression models including sex and either hour, weekday or month were estimated as follows: sex + hour, sex + weekday and sex + month. The effects are presented by giving relative risk estimates and 95% confidence intervals.

This study was proved ethically feasible by the Ethics Committee of the Medical Faculty of Cerrahpasa at Istanbul University (Nr. B.30.2.IST.0.30.90.00/10046).

## Results

In the present study, we analysed retrospectively 2232 records of young suicide attempters. The majority of the study sample were females (81.6%, N = 1822 females vs. 18.4%, N = 410 males). Nearly all study subjects 99.8% (N = 2227) were aged between 14 and 25.

There was no sex difference in the methods of suicide attempts. Female as well as male young patients chose low-risk methods for their suicide attempts [13]. The majority of the suicide attempters chose drug intoxication (94.4%) followed by injury with sharp objects (1.4%). More females (95.1%) than males (91.5%) chose drug intoxication for their suicide attempts. In contrast, more males (2.2%) than females (1.2%) made a suicide attempt by injury with a sharp object.

In 4.4% of cases the information about the day, and in 12.3% of cases the information about the time of day of the suicide attempt, were missing. In all regression models the effect of sex as well as the effects of hour, and weekday were significant (p < 0.001).

**Fig. 1:**
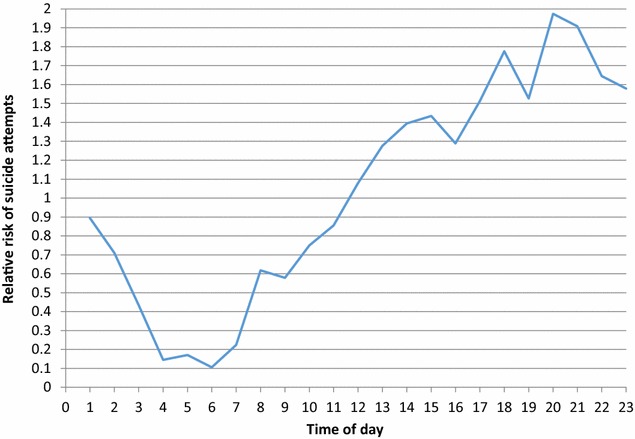
Risk of suicide attempts associated with time of day (Reference hour 0:00 h)

**Fig. 2:**
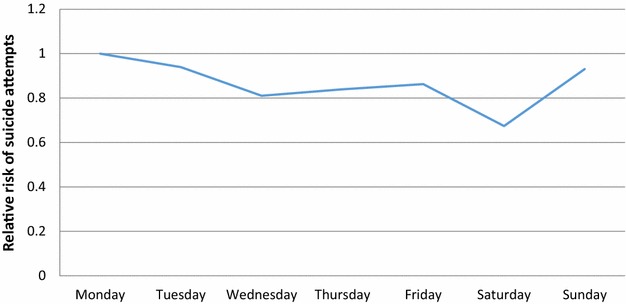
Risk of suicide attempts associated with weekday (Reference day: 1-Monday)

**Fig. 3:**
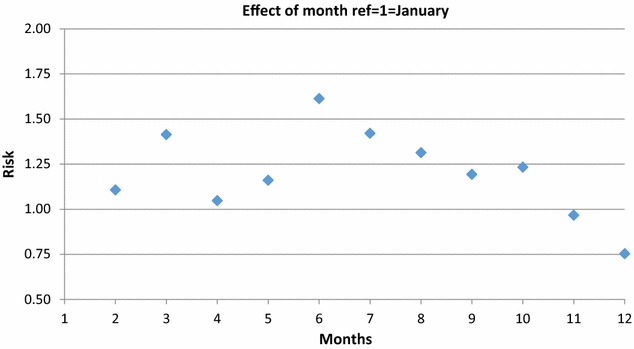
Risk of suicide attempts associated with months

Corresponding relative risk estimates and their confidence intervals are shown in Tables 1, 2 and Figs. 1, 2, 3.

**Table 1:** Sex differences by risk of suicide attempts associated with time of day

Parameter	Level	Relative risk	Lower 95%CI	Upper 95%CI
Hour	0:00 = Ref	1	–	–
	1:00	0.89	0.64	1.25
	2:00	0.71	0.50	1.02
	3:00	0.43	0.29	0.66
	4:00	0.14	0.08	0.28
	5:00	0.17	0.09	0.31
	6:00	0.11	0.05	0.22
	7:00	0.22	0.13	0.38
	8:00	0.62	0.43	0.90
	9:00	0.58	0.40	0.85
	10:00	0.75	0.53	1.07
	11:00	0.86	0.61	1.20
	12:00	1.08	0.78	1.49
	13:00	1.28	0.94	1.74
	14:00	1.39	1.03	1.89
	15:00	1.43	1.06	1.94
	16:00	1.29	0.95	1.75
	17:00	1.51	1.12	2.04
	18:00	1.78	1.33	2.37
	19:00	1.53	1.13	2.05
	20:00	1.97	1.49	2.62
	21:00	1.91	1.43	2.54
	22:00	1.64	1.23	2.20
	23:00	1.58	1.18	2.12
Sex	Female = ref	1	–	–
	Male	0.22	0.19	0.25

**Table 2:** Sex differences by risk of suicide attempts associated with weekdays and months

Parameter	Level 1	Relative risk	Lower 95%CI	Upper 95%CI
(a)				
Weekday	Monday = Ref	1	–	–
	Tuesday	0.94	0.82	1.08
	Wednesday	0.81	0.70	0.94
	Thursday	0.84	0.73	0.97
	Friday	0.86	0.75	1.00
	Saturday	0.67	0.58	0.79
	Sunday	0.93	0.81	1.07
Sex	Female = ref	1	–	–
	Male	0.34	0.31	0.38
(b)				
Month	January = Ref	1	–	–
	February	1.11	0.86	1.43
	March	1.41	1.11	1.80
	April	1.05	0.81	1.36
	May	1.16	0.90	1.50
	June	1.61	1.27	2.04
	July	1.42	1.11	1.81
	August	1.31	1.03	1.68
	September	1.19	0.93	1.54
	October	1.23	0.96	1.58
	November	0.97	0.74	1.26
	December	0.75	0.57	1.00
Sex	Female = ref	1	–	–
	Male	0.22	0.20	0.26

As we can see in Fig. 1, we took midnight as reference, so the results show that starting from the midday the risk for a suicide attempt is increasing. The risk for a suicide attempt is highest during the evening (17:00–23:00) and lowest around 4:00 and 7:00 a.m. There were no significant sex differences (p = 0.44) with regard to time of day of suicide attempts.

We took Monday as a reference, so our results show that the risk for a suicide attempt is lower than Monday during the other weekdays (Tuesday–Sunday). There were no significant sex differences (p = 0.69) concerning the day of the week of suicide attempts.

In Fig. 3 we took January as a reference, the risk for a suicide attempt is highest in the beginning of spring (March) and in the summer months (June until August). However, the risk of suicide attempt is lower in November and December. There were no significant sex differences concerning the months of the suicide attempts (p = 0.19).

## Discussion

The results of the present study show that there is a monthly, diurnal and circadian rhythm of suicide attempts among youth. First most suicide attempts of young people occurred in the evening up until midnight. This result can be observed in both sexes. Second, suicide attempts most frequently occurred at the beginning of the week in both sexes, independent of season.

Concordant to these findings, other studies report that suicide attempts most frequently occur between the evening and midnight [8, 16]. Given that studies on reasons for suicide attempts and self-harm in youth report interpersonal conflicts to be the most common trigger for the act [2, 26, 27], we assume that particularly in the evening time, when family members come together, conflicts between family members may contribute to suicide attempts. This is underscored by the fact, that intra-familial conflicts are the most common reason for suicide attempts in Turkish youth [28].

Jessen et al. found that half of all suicide attempts occurred in the evening or early part of the night [29]. Furthermore, the study of Valtonen et al. suggests that people contact health-care services mostly during late evening and midnight hours [17]. Our findings suggest, that help lines and health-care services for young people should be available especially in the late evening time [30].

Consistent with our results, previous studies reported a higher incidence of suicidality at the beginning of the week [15, 19] and low incidence of suicidal behaviour on Saturday [31].

This finding can reflect difficulties among youth when returning to structured and routine lifestyle after the weekend. Problematic work situations may be more wearing and difficult at the beginning of the week. We also assume that school stress can increase the incidence of suicide attempts on Sunday and Monday, as young people can have more fear and stress to confront school-associated problems again after the weekend. School-related problems have already been reported to have an association with suicidal behaviours [32–34]. Additionally, the peak of suicide attempts at the beginning of the week can also be explained by the “broken promise effect”, as the week-end is often perceived as a positive recreational period of the week, which sometimes may promise more than it delivers [12, 35].

In most cases, suicide attempts are triggered by interpersonal problems among family members, which may be highest at the week-end [30]. Furthermore, persons with psychiatric disorders, being a common risk factor for suicide attempts, are supposed to have more negative feelings like personal failure or isolation at the beginning of the week [36].

An association between months of the year and suicide attempts [17, 37] as well as completed suicides [38] has already been reported. Confirming previous studies, our present study shows there was a peak in suicide attempts during the summer months [39] and a nadir in suicide attempts was present during winter months [17]. We suppose that the “Broken-Promise Effect” arises more in the summer months. As Turkey has a collectively oriented community, and the family including relatives and friends are getting together more during holidays in the summer months, intra-familial problems can arise more frequently, which leads to the “Broken-Promise Effect”, when the expectation of a positive event evokes frustration, if it promises more than it delivers [35]. We also suppose that the stress because of public exams in Turkey may play a role in suicide attempts of young people in spring and summer months. In Turkey there are public exams for entrance to the university and for public employment, which are conducted annually in spring and summer months. The peak in summer months could be due to the stress caused by these public exams [40].

In line with international studies [8, 41–46], also in this study sample the majority (81.6%) of suicide attempters were females. Male suicide attempters are generally described to choose more lethal methods, which more often end fatally [47] and therefore are not recorded as suicide attempts.

## Conclusion

As suicidal behaviour is an increasingly important public health issue, the results of the present study can help to understand psychosocial mechanisms of suicide attempts better and aid in developing adequate preventive measures to reduce suicidal behaviour among young people [1]. Given the fact that suicide attempts among youth follow a diurnal and circadian pattern (evenings and weekends) contrary to the usual availability of services, the development of adequate treatment settings and hotlines for youth with suicidal behaviour and their family members should be taken seriously.

### Limitations

The present study is based on a retrospective analysis. During the treatment at emergency units of hospitals, the forms about suicide attempts were not filled out in complete detail, therefore many data like birthday and exact age of the suicide attempters were missing. Our results are based on existing data. Missing data is a big problem because of selection bias, and the reason for the lacking information is not clear.

Therefore, the present findings cannot be extended to the general population and need to be replicated in a sample with more detailed characteristics of young suicide attempters.

We assume that the number of unrecorded suicide attempts in Istanbul may be much higher than the registered, given that suicide attempts which do not require any medical treatment are not recorded in this system.

As the forms about suicide attempts were filled out by different medical staff, there may be reliability problems.

## References

[1] Greydanus DE, Calles JJ (2007). Suicide in children and adolescents. Prim Care.

[2] Schmidtke A, Bille-Brahe U, DeLeo D, Kerkhof A, Bjerke T, Crepet P (1996). Attempted suicide in Europe: rates, trends and sociodemographic characteristics of suicide attempters during the period 1989–1992. Results of the WHO/EURO Multicentre Study on Parasuicide. Acta Psychiatr Scand.

[3] World Health Organisation: Child and adolescent mental health. http://www.who.int/mental_health/women_children/child_adolescent/en/index.html. 2014. Accessed 15 May 2015.

[4] Blum RW, Nelson-Mmari K (2004). The health of young people in a global context. J Adolesc Health.

[5] Mittendorfer-Rutz E, Rasmussen F, Wasserman D (2004). Restricted fetal growth and adverse maternal psychosocial and socioeconomic conditions as risk factors for suicidal behaviour of offspring: a cohort study. Lancet.

[6] Varnik A, Kolves K, Allik J, Arensman E, Aromaa E, van Audenhove C (2009). Gender issues in suicide rates, trends and methods among youths aged 15–24 in 15 European countries. J Affect Disord.

[7] Wasserman D, Cheng Q, Jiang G (2005). Global suicide rates among young people aged 15–19. World Psychiatry..

[8] Doganay Z, Sunter AT, Guz H, Ozkan A, Altintop L, Kati C (2003). Climatic and diurnal variation in suicide attempts in the ED. Am J Emerg Med.

[9] Lewinsohn PM, Rohde P, Seeley JR, Baldwin CL (2001). Gender differences in suicide attempts from adolescence to young adulthood. J Am Acad Child Adolesc Psychiatry.

[10] Nock MK, Borges G, Bromet EJ, Cha CB, Kessler RC, Lee S (2008). Suicide and suicidal behavior. Epidemiol Rev.

[11] American Foundation for Suicide Prevention 2015 Facts and figures. https://www.afsp.org/understanding-suicide/facts-and-figures. Accessed 10 Oct 2015.

[12] Akkaya-Kalayci T, Popow C, Waldhor T, Ozlu-Erkilic Z (2015). Impact of religious feast days on youth suicide attempts in Istanbul, Turkey. Neuropsychiatrie.

[13] Akkaya-Kalayci T, Popow C, Winkler D, Bingol RH, Demir T, Ozlu Z (2015). The impact of migration and culture on suicide attempts of children and adolescents living in Istanbul. Int J Psychiatry Clin Pract..

[14] Skala K, Bruckner T (2014). Beating the odds: an approach to the topic of resilience in children and adolescents. Neuropsychiatrie.

[15] Beauchamp GA, Ho ML, Yin S (2014). Variation in suicide occurrence by day and during major American holidays. J Emerg Med.

[16] Polewka A, Kroch S, Chrostek Maj J (2004). Suicidal behavior and suicide attempts in adolescents and young adults—epidemiology, risk factors, prevention and treatment. J. Przegl Lek.

[17] Valtonen H, Suominen K, Partonen T, Ostamo A, Lonnqvist J (2006). Time patterns of attempted suicide. J Affect Disord.

[18] Modan B, Nissenkorn I, Lewkowski SR (1970). Suicide in a heterogeneous society. Br J Psychiatry.

[19] Corcoran P, Reilly M, Salim A, Brennan A, Keeley HS, Perry IJ (2004). Temporal variation in Irish suicide rates. Suicide Life Threat Behav.

[20] Law CK, Leung CM (2012). Temporal patterns of charcoal burning suicides among the working age population in Hong Kong SAR: the influence of economic activity status and sex. BMC Public Health..

[21] Caracciolo S, Manfredini R, Gallerani M, Tugnoli S (1996). Circadian rhythm of parasuicide in relation to violence of method and concomitant mental disorder. Acta Psychiatr Scand.

[22] Pandit A (2004). Circadian rhythm variation in attempted suicide by deliberate self-poisoning, and in completed suicide, in Central Nepal. Biol Rhythm Res.

[23] Lukaschek K, Baumert J, Erazo N, Ladwig KH (2014). Stable time patterns of railway suicides in Germany: comparative analysis of 7,187 cases across two observation periods (1995–1998; 2005–2008). BMC Public Health..

[24] Preti A, Miotto P (2001). Diurnal variations in suicide by age and gender in Italy. J Affect Disord.

[25] van Houwelingen CA, Beersma DG (2001). Seasonal changes in 24-h patterns of suicide rates: a study on train suicides in The Netherlands. J Affect Disord.

[26] Deveci A, Taskin EO, Erbay Dundar P, Demet MM, Kaya E, Ozmen E (2005). The prevalence of suicide ideation and suicide attempts in Manisa City Centre. Turk Psikiyatri Derg.

[27] Lee MT, Wong BP, Chow BW, McBride-Chang C (2006). Predictors of suicide ideation and depression in Hong Kong adolescents: perceptions of academic and family climates. Suicide Life Threat Behav.

[28] Yalaki Z, Tasar MA, Yalcin N, Dallar Y (2011). Cocuk ve genclik dönemindeki özkiyim girisimlerinin degerlendirilmesi. Ege Tip Dersigisi..

[29] Jessen G, Jensen BF, Arensman E, Bille-Brahe U, Crepet P, De Leo D (1999). Attempted suicide and major public holidays in Europe: findings from the WHO/EURO multicentre study on parasuicide. Acta Psychiatr Scand.

[30] Jessen G, Andersen K, Arensman E, Bille-Brahe U, Crepet P, De Leo D (1999). Temporal fluctuations and seasonality in attempted suicide in Europe. Findings from the WHO/EURO multicentre study on parasuicide. Archiv Suicide Res.

[31] Nishi M, Miyake H, Okamoto H, Goto Y, Sakai T (2000). Relationship between suicide and holidays. Epidemiology.

[32] Dervic K, Akkaya-Kalayci T, Kapusta ND, Kaya M, Merl E, Vogel E (2007). Suicidal ideation among Viennese high school students. Wien Klin Wochenschr.

[33] Dervic K, Gould MS, Lenz G, Kleinman M, Akkaya-Kalayci T, Velting D (2006). Youth suicide risk factors and attitudes in New York and Vienna: a cross-cultural comparison. Suicide Life Threat Behav.

[34] Ranney ML, Patena JV, Nugent N, Spirito A, Boyer E, Zatzick D (2016). PTSD, cyberbullying and peer violence: prevalence and correlates among adolescent emergency department patients. Gen Hosp Psychiatry.

[35] Gabennesch H (1988). When promises fail: a theory of temporal fluctuations in suicide. Soc Forces.

[36] Erazo N, Baumert J, Ladwig KH (2004). Sex-specific time patterns of suicidal acts on the German railway system. An analysis of 4003 cases. J Affect Disord.

[37] Mergl R, Havers I, Althaus D, Rihmer Z, Schmidtke A, Lehfeld H (2010). Seasonality of suicide attempts: association with gender. Eur Arch Psychiatry Clin Neurosci.

[38] Rocchi MB, Sisti D, Cascio MT, Preti A (2007). Seasonality and suicide in Italy: amplitude is positively related to suicide rates. J Affect Disord.

[39] Polewka A, Szkolnicka B, Targosz D, Groszek B, Kroch S, Chrostek Maj J (2004). Fluctuations and seasonality in suicidal attempts. Przegl Lek.

[40] ÖSYM 2010 Öğrenci Seçme ve Yerleştirme Merkezi. 2010 Yılı Sınav Takvimi. http://www.osym.gov.tr/belge/1-12296/2010-sinav-takvimi.html. Accessed 10 Feb 2009.

[41] Cam B (2012). Suicide attempt, sociodemographic characteristics, and seasonality. Klinik Psikofarmakoloji Bülteni..

[42] Elisei S, Verdolini N, Anastasi S (2012). Suicidal attempts among Emergency Department patients: one-year of clinical experience. Psychiatr Danub..

[43] Monnin J, Thiemard E, Vandel P, Nicolier M, Tio G, Courtet P (2012). Sociodemographic and psychopathological risk factors in repeated suicide attempts: gender differences in a prospective study. J Affect Disord.

[44] Naidoo SS, Schlebusch L (2013). Sociodemographic and clinical profiles of suicidal patients requiring admission to hospitals south of Durban. S Afr Fam Pract..

[45] Rancic N, Ignjatovic Ristic D, Radovanovic S, Kocic S, Radevic S (2012). Sociodemographic and clinical characteristics of hospitalized patients after suicide attempt: a twenty-year retrospective study. Med Glas (Zenica)..

[46] Aydin A, Gulec M, Boysan M, Selvi Y, Selvi F, Kadak MT (2013). Seasonality of self-destructive behaviour: seasonal variations in demographic and suicidal characteristics in Van, Turkey. Int J Psychiatry Clin Pract..

[47] Mann JJ (2002). A current perspective of suicide and attempted suicide. Ann Intern Med.

